# Increasing Fecal Immunochemical Test Return Rates by Implementing Effective “Reminder to Complete Kit” Communication With Participants: A Quality Improvement Study

**DOI:** 10.7759/cureus.25169

**Published:** 2022-05-20

**Authors:** Sameer Prakash, Nooraldin Merza, Omid Hosseini, Haven Ward, Tarek Mansi, Michelle Balducci, Deborah Trammell, Brenda Hernandez, Izi Obokhare

**Affiliations:** 1 Internal Medicine, University of Iowa Hospitals and Clinics, Iowa City, USA; 2 Internal Medicine, Memorial Hermann The Woodlands Hospital, Spring, USA; 3 Internal Medicine, Wayne State University School of Medicine, Detroit, USA; 4 School of Medicine, Texas Tech University Health Sciences Center, Lubbock, USA; 5 Internal Medicine, Texas Tech University Health Sciences Center, Amarillo, USA; 6 General and Colorectal Surgery, Texas Tech University Health Sciences Center, Amarillo, USA

**Keywords:** colorectal cancer, quality improvement research, colon cancer prevention, early screening, disease surveillance and notification

## Abstract

Background

The incidence of colorectal cancer (CRC) in the United States is increasing. It remains the second leading cause of cancer death in the United States for men and women combined, mainly due to underutilization of screening methods. The American Cancer Society now recommends that adults aged 45 years and older with an average risk of CRC undergo regular screening with either a high-sensitivity stool-based test or structural (visual) examination, depending on patient preference and test availability. The primary objective of this quality improvement project was to determine if reminder methods, such as telephone or letter reminders, increased the return rate of fecal immunochemical tests (FIT) for CRC screening.

Methodology

At public outreach events and daily clinics in the West Texas Panhandle area, participants in the GET FIT program were provided with FIT kits after completing the education on CRC. Participants who fit the inclusion criteria and had received a FIT kit from the program were included. They were instructed on how to perform the test and mail it back. Participants who did not return the completed kits within two weeks were reminded either through (1) a reminder letter, (2) telephone, or (3) a combination of letter reminder and telephone call every two weeks (±three days) for 60 days or five attempts to contact. We de-identified and analyzed the FIT kit return data from April-September 2019 before analyzing these reminder methods. We then calculated the change in return rates from October 2019 to March 2020. Our goal was to increase the FIT return rates by 25% compared to the baseline return rate.

Results

The pre-intervention return rate of kits for April-September 2019 was 61.52%, and the post-intervention return rate for October 2019-March 2020 was 71.85%. This rate was equal to an approximately 16.79% increase in return rates that was statistically significant (p < 0.01). There was a significant difference in the method of reminder between the two groups, but no significant differences in gender and race/ethnicity between the two groups. There was a significant difference in return rates between race/ethnicities in the October-March cohort with black and Hispanic participants having the highest return rates of 82.3% and 77.25%, respectively.

Conclusions

FIT remains one of the primary options for CRC screening. Due to its lower cost and noninvasiveness, FIT was offered to patients at average risk. We demonstrated an increase in return rates, although we did not meet our target return rate goal for this project. This study was limited due to a gradual increase in coronavirus disease 2019 (COVID-19) cases and a subsequent shift and conversation of ongoing research into COVID-19.

## Introduction

The incidence of colorectal cancer (CRC) has significantly increased in the last few decades with the onset of earlier diagnosis becoming more pervasive. Screening rates remain lower than the recommended 70% and 80% targets, especially among low-income (47%), uninsured (25%), African American (59%), Asian (52%), Native American (48%), and Hispanic (47%) populations [1]. Fecal immunochemical test (FIT) remains a viable option for patients as an alternative with lower cost and ease of testing. The American College of Gastroenterology (ACG) 2021 guidelines recommend CRC screening starting at the age of 45-50 using colonoscopy and FIT as the primary screening modalities [2]. Recommendations by the U.S. Preventative Task Force (USPSTF) [3] and the Centers for Disease Control and Prevention (CDC) recommend annual FIT followed by colonoscopy for any positive tests. The American Cancer Society (ACS) also shared similar recommendations [4]. Financial barriers to uninsured and underinsured patients persist in colorectal screening, especially with the gold standard of screening being the colonoscopy.

On comparing chemical and immunochemical tests, the FIT showed greater sensitivity, specificity, and predictive values in detecting colorectal bleeding and moderate concordance with the colonoscopy. In a composite review of five studies, the average sensitivity and specificity of FIT for CRC were 93% and 95%, respectively, and moderate accuracy for advanced neoplasia. In federal health centers, approximately 50-60% of patients with a resultant positive FIT go on to have a follow-up colonoscopy [5]. In a retrospective study of 40,000 patients, risks were significantly higher when colonoscopy was delayed by more than six months for any form of CRC and further increased after 12 months [6]. The cost of increasing years of life gained (YLG) was studied in a population-level screening model in a rural population. This showed a cost-benefit analysis suggesting that the screening program begins to yield positive net benefits at the stage when recipients undergo colonoscopy, suggesting that this is the key step for behavioral intervention and intensified outreach [7].

Comparison of telephone versus mail-based or email/internet-based communication has been seldom studied in the literature on methods to improve colorectal screening. Studies involving intervention clinics have demonstrated higher proportions of patient involvement and improved rates of FIT completion. Electronic health record embedded implementation of mailed reminders improved rates of both FIT completion rates (13.9 vs. 10.4% difference) and CRC screening (18.3 vs. 14.5% difference) compared to usual care clinics [8]. Gupta et al. showed almost twice as effective screening with invitations to FIT versus colonoscopy in underserved patients not up to date on CRC screening and telephone follow-up effectively increasing screening [9]. Miller et al. found that by involving digital health intervention through a mobile app at Wake Forest, screening rates were 30% in the group using the app versus 15% in the usual care group [10,11].

Screening rates for CRC screening continue to remain at an all-time low in the United States and worldwide. The more patients are reminded and have close follow-ups with their providers, the better opportunity there is to prevent this deadly disease. We hypothesize that there will be an increase in the FIT kit return rate of approximately 25% through a combination of telephone and letter reminder methods.

## Materials and methods

This study was a retrospective study conducted through the GET FIT program. Participants were enrolled in the program at public outreach events and daily internal and external outpatient clinics. They were informed about the utility of FIT as a method of CRC screening. Included patients were required to have a physical address and phone number listed on the enrollment form and must not have had their kit returned in the previous two weeks. We excluded patients who returned FIT kits earlier than two weeks from the dispensed date and/or who did not have contact information listed on the enrollment form.

At public outreach events and daily clinics, participants in the GET FIT program were provided with the FIT kit after completing the education on CRC. Participants were instructed on how to perform the test and mail the completed test back. Participants who did not return the completed FIT kit within two weeks were randomized to a 1:1 protocol consisting of (1) telephone reminders and (2) a combination of both telephone calls and letters, every two weeks (±three days) for a period of 60 days or five attempts to contact.

We collected data on demographic information of patients, including gender, race, date(s) of the telephone call, date of the reminder letter, whether the kit was distributed at the clinic or public outreach event, and if the FIT kit was returned. The data gathered was de-identified and entered into a password-protected computer database for analysis. We analyzed FIT return rates from patients for six months from April to September 2019 before instituting the reminder protocol and analyzing the FIT return rates with the intervention from October 2019 to March 2020. Our post-intervention analysis through March was hindered due to the coronavirus disease 2019 (COVID-19) pandemic and the lack of personnel unable to collect FIT kits. We did not compare and analyze the letter-only reminder method with the other reminder methods due to the lack of patients in this arm in both time periods. The characteristics of the study population between the two time frames of April 2019 to September 2019 and October 2019 to March 2020 were compared using the chi-square analysis.

## Results

From April 1, 2019, to September 30, 2019, 283 of 460 total participants returned FIT kits for an overall return rate of 61.52%. There were more females than males (66.96%) and more Hispanic participants than other races (48.37%). From October 1, 2019, to March 15, 2020, there were a total of 508 participants, with 315 (62%) females and 193 (38%) males. In total, 365 FIT tests were returned (71.85%), and 143 FIT tests were not returned (28.1%). There was a statistically significant difference in return rates between the two time periods (p < 0.01). The characteristics of the study population between the two time frames of April 2019 to September 2019 and October 2019 to March 2020 were compared using the chi-square analysis. No significant differences in sex and race/ethnicity between the two groups were found (p = 0.086 and p = 0.23, respectively). There was a significant difference in the method of reminder between the two groups resulting in a p-value of <0.0001. The time frame from April 2019 to September 2019 had 40.4% in the no reminder group, 27.7% in the phone-only group, and 32.4% in the combination group. For the time frame from October to March, 55.9% were in the no reminder group, 21.2% in the phone group, and 22.9% in the combined group. Characteristics of the study population are listed in Table 1.

**Table 1:** Characteristics of the study population. Differences between time frames were calculated using the chi-square test. There were no significant differences in sex and race/ethnicity between the two time frames. There was a significant difference in the method of reminder reflecting the changes implemented. Two subjects were missing data about sex (noted with *, ~0.05%).

Variable	Reminder: April 2019 to September 2019 (N = 460)	Reminder: October 2019 to March 2020 (N = 499)	P-value
Sex			0.086
Male	150 (32.6 %)*	190 (38.1%)	-
Female	308 (66.9%)*	309 (61.9%)	-
Race/Ethnicity			0.23
White	200 (43.5%)	199 (39.9%)	-
Hispanic	223 (48.5%)	255 (51.1%)	-
Black	21 (4.6%)	17 (3.4%)	-
Other	16 (3.4%)	28 (5.6%)	-
Method of reminder			<0.00001
No reminder	186 (40.4%)	279 (55.9%)	-
Phone	125 (27.2%)	106 (21.2%)	-
Combination	149 (32.4%)	114 (22.9%)	-

The average length of time between kit issued and kit returned was approximately 12 days, suggesting that follow-up calls should begin two weeks from the kit issued date. Of the 279 who received no reminders of any kind, 88.53% returned them and 11.47% did not return them. Overall, 74.49% of kits in this group were returned before the two-week follow-up window. Therefore, these participants were excluded from the analysis of patients subjected to reminder methods of telephone, letter, or a combination of telephone/letter methods. We excluded the letter-only method from the analysis due to low numbers. Of the 106 who received reminders by phone call, 81 (76.42%) returned them and 25 (23.58%) did not return them. In total, 64 (79%) participants did so after the first call, followed by an additional 11 (13.6%) after two calls, and six (7.4%) needed three or more calls to return their kit.

**Table 2:** Comparison of studies examining system-based interventions and adherence to screening. CRC: colorectal cancer; FIT: fecal immunochemical test

Author	Main findings
Myers et al. (2007)	Intervention groups: mail with education/FIT kit reminder, paging intervention, and combination of mailed/page/telephone reminders Two-year CRC screening rates were significantly higher in the three intervention groups than in the control group and did not differ significantly among intervention groups (33% in the control group, 46%, 44%, and 48% in intervention groups 2–4, respectively) [11]
Singal et al. (2016)	FIT outreach adherence with mailed kit and telephone reminders was increased compared to usual primary care (58% vs. 29.6%) and higher than colonoscopy adherence (42.2%) [12]
Inadomi et al. (2021)	Multicomponent interventions compared to single-component interventions have been shown to be more effective in increasing CRC screening, (RR, 1.92 [95% CI, 1.69-2.19] vs. RR, 1.43 [95% CI, 1.19-1.71]) [13,14]
Dougherty et al. (2018)	Meta-analysis of US-based interventions in clinical settings examined varying levels of follow-up reminders and/or navigation to usual care. Increased screening completion in year two as well as an increased rate of complete adherence to screening guidelines through three years [13]
Cha et al. (2011)	Telephone reminders in addition to mail notification of positive FIT results increased the acceptance rate of colonoscopy compared to mail notification alone (p = 0.046)

Of the 114 participants who had a combination of phone calls and letter reminders, 27.19% returned them and 72.81% did not return them. Similar to the pre-intervention period, people in the combination method group had a much lower return rate (27.19%) than those who received no reminders (88.53%) or received reminders by phone only (76.42%).

**Figure 1:**
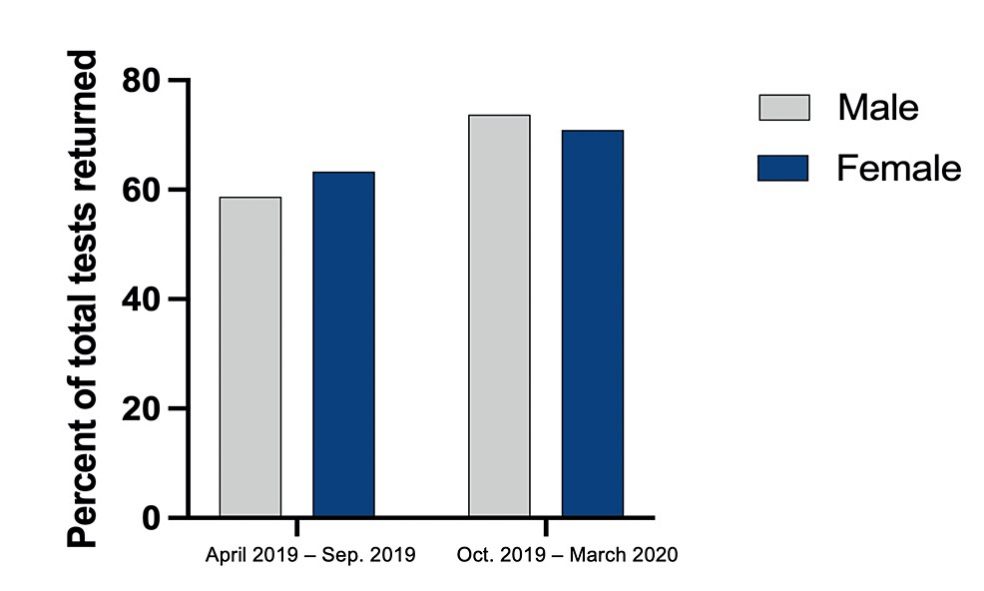
Test return rates by sex. Using the chi-squared test, we computed differences in return rates for both time frames. From April 2019 to September 2019, there was no significant difference between return rates when split by sex (males = 58.67%, females = 63.31%, p = 0.36). Despite the slight increase in return rates, there was no significant difference between males and females from October 2019 to March 2020 (males = 73.68%, females = 70.87%, p = 0.54).

**Figure 2:**
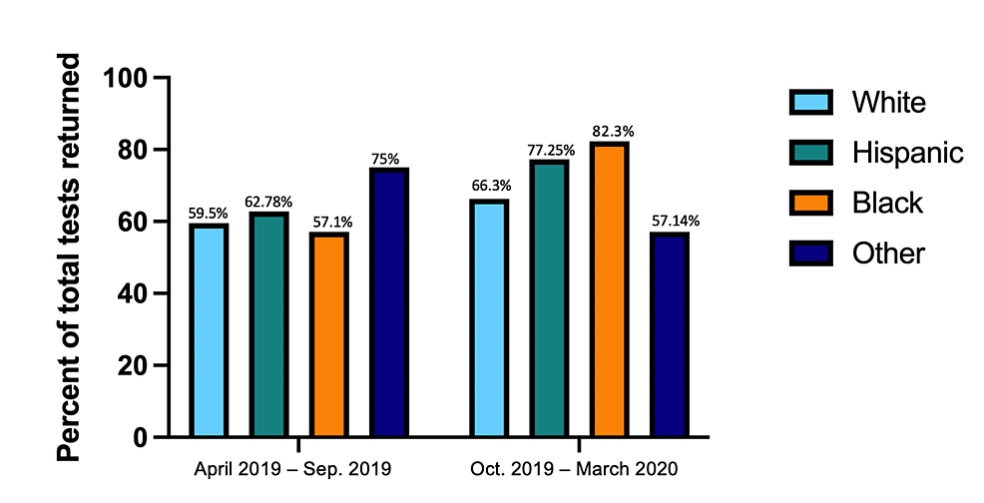
Test return rates split by race/ethnicity. Between April 2019 to September 2019, there was no significant difference between return rates when split by race/ethnicity (White = 59.5%, Hispanic = 62.78%, Black = 57.1 %, other = 75.00%, p = 0.54). There were statistically significant differences in return rates from October 2019 to March 2020 with Black and Hispanic participants have the highest return rates (White = 66.3%, Hispanic = 77.25%, Black = 82.3%, other = 57.14 %, p = 0.014). Both time frames were compared using the chi-square test.

**Figure 3:**
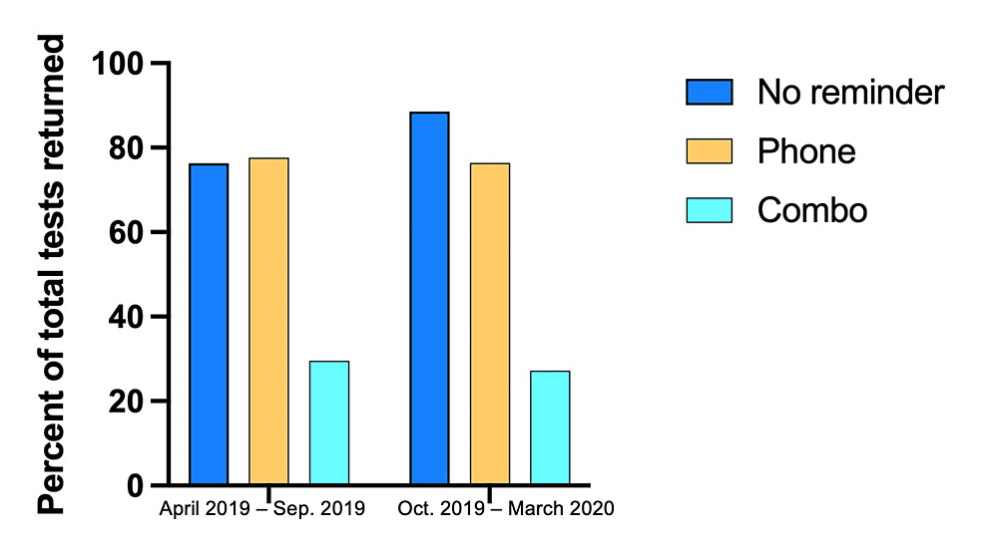
Return rates by reminder method. Both time frames have statistically different return rates when comparing reminder method (chi-square, p < 0.0001 for both frames). No reminder and phone had the highest return rates compared to the combination method (April 2019 to September 2019: no reminder = 76.3%, phone = 77.6%, combination = 29.5%; October 2019 to March: no reminder = 88.5%, phone = 76.4%, combination = 27.19%).

Return rates between sex in both time frames were compared using the chi-square tests. From April 2019 to September 2019, there was no significant difference between sex resulting in a p-value of 0.36. Return rates in that group for males were 58.67% and 63.31% for females. In regards to October 2019 to March 2020 group, return rates were 73.68% for males and 70.87% for females without a significant difference between sexes (p = 0.54) (Figure 1). We also calculated the return rate of FIT kits in new and repeat patients. In new and repeat patients (n = 309, n = 149, respectively), 181 new (58.58%) and 102 repeat patients (68.46%) returned kits. When comparing the rates of return between race/ethnicities, no significant differences were found in the April to September group. There was a significant difference in return rates between race/ethnicities (p = 0.014) in October to March group with black and Hispanic participants having the highest return rates of 82.3% and 77.25%, respectively. Rates of return split by race/ethnicity can be seen in Figure 2. The chi-square test comparing the return rates by the reminder method resulted in a significant difference of p < 0.0001 for both time frames. No reminder and phone had the highest return rates compared to the combined method (Figure 3).

## Discussion

The importance of timely screening for CRC can be improved through patient reminders and patient education. In our study, we hypothesized that a formal reminder system of telephone plus letter reminder methods would increase FIT return rates versus telephone and letter methods only. We did not demonstrate the hypothesized 25% increase in FIT return rates after instituting this reminder system after April-September 2019 and October 2019-March 2020. Previous studies have documented that telephone methods as an additional or solitary reminder may not increase FIT return rates. Levy et al. found no significant difference in FIT return rates with mailed education alone (45.2%) versus mailed education with telephone reminders (48.7%, p = 0.498), but did find an increase in the return rate of questionnaires assessing educational materials [12]. In our study, we found that the telephone method was very effective in the return of FIT kits, with more than 79% of participants returning their kits after the first call itself. Both time frames have statistically different return rates when comparing the reminder method with the telephone method (77.6%, 76.4%) and no reminder (76.3%, 88.5%) having the highest return rates. Furthermore, in the post-intervention group of October-March, approximately 75% of kits were returned before the two-week follow-up window. Surprisingly, most participants (72.8%) did not return kits in the combination of phone calls and letter reminders. This may suggest that having multiple reminder methods may deter participants from returning kits and spacing out reminders may be more practical.

One would expect adherence to screening to be increased through targeted and repeated reminders. Table 2 summarizes the findings of several studies examining intervention reminder methods and the effect on CRC screening rates. Studies have shown an increase in cancer screening rates through interventions versus no reminders [13,14], Among the intervention groups, there was not a significant difference in screening rates. Furthermore, the combination of intervention methods also potentially increases screening cancer rates rather than single-component interventions, especially with the acceptance rate of colonoscopy [15-17]. However, our study differed from other studies in that individuals in the combination method group had a much lower return rate than those who received no reminders or received reminders by phone only. This may suggest that patients may be less likely to return FIT kits if they receive multiple reminders and that patients may prefer single-component intervention reminders.

There are significant gender and racial disparities in cancer screening. Gender disparities in cancer screening are most likely multifactorial, including reasons such as stigma from the procedure, financial burden, and lack of education about the reasons for screening. The incidence of CRC is increased among men compared to women as well as recurrence and survival [18], yet women were less likely to undergo screening despite accounting for insurance status, income, and education level [19]. In our study, we found no significant differences among gender in both time periods despite a slight increase in return rates. A more comprehensive educational gender-specific approach may be needed to account for hesitancy in screening by gender. As mentioned, screening in minority populations remains well below the average target rate of 70% and 80%, especially with the Hispanic population lowest at 47%. In our study, we found that from October 2019-March 2020, the Hispanic and African American populations had the highest return rates and were statistically significant (Hispanic = 77.25%, Black = 82.3%, p = 0.014). This shows that a systematic reminder method can benefit underserved populations. Response rates in underserved populations are closely linked to the connection and contact with healthcare providers. There are multiple strong socio-economical barriers that would need to be overcome to increase the screening rates in underserved populations such as financial, transportation, education, social stigma associated with testing, and many more other minor confounding factors. Our limitation for this study was that our sample size was low in these populations.

Our results should be interpreted considering the limitations of the study. One limitation was the lack of participants who only received a reminder letter, with nine individuals in April-September 2019 and no individuals in October 2019-March 2020. Instituting this method alone may have been beneficial as it has been documented in the literature that mailed outreach can increase CRC screening, especially in underserved populations. A future study is needed to evaluate the impact that public outreach events and primary care physicians have in improving screening, as we noted a significant increase in return rates after a public screening event in October 2019. Another limitation was that our sample size was low for evaluating the impact of reminder methods in populations by ethnicity.

## Conclusions

In summary, our study shows that the telephone reminder itself is effective in increasing FIT return rates. Multiple reminders may negatively impact FIT return rates, and thus spacing out reminders may be helpful. There were no significant differences regarding gender in FIT return rates and Hispanic and African American participants were more likely to return FIT kits. Targeting underserved populations, especially in rural areas, for CRC screening is crucial in meeting the proposed target rates.
